# Sex in the City: Breeding Behavior of Urban Peregrine Falcons in the Midwestern US

**DOI:** 10.1371/journal.pone.0159054

**Published:** 2016-07-15

**Authors:** Isabel C. Caballero, John M. Bates, Mary Hennen, Mary V. Ashley

**Affiliations:** 1 Department of Biological Sciences, University of Illinois at Chicago, Chicago, Illinois, United States of America; 2 Department of Biology, Texas A&M University, College Station, Texas, United States of America; 3 Integrative Research Center, Field Museum of Natural History, Chicago, Illinois, United States of America; 4 Gantz Family Collection Center, Field Museum of Natural History, Chicago, Illinois, United States of America; University of Padova, ITALY

## Abstract

Peregrine falcons (*Falco peregrinus*) were extirpated from most of the continental United States by widespread use of the pesticide DDT in the 1960s. Populations have rebounded with banning of the pesticide and successful implementation of captive breeding and hacking programs. An essentially new population of Midwestern peregrines now exists that is comprised almost entirely of urban-nesting birds. The new population is considered to be of mixed ancestry, occurs at relatively high densities, and has nest sites in close proximity, factors that could influence breeding behaviors including mate fidelity, nest-site fidelity, extra-pair paternity, and natal dispersal. We investigated these behaviors using a combination of field observations and DNA microsatellite genotyping. Data for eleven microsatellite DNA markers, including eight newly developed for the species, were analyzed from a total of 350 birds from nine Midwestern cities, representing 149 broods collected at 20 nest sites. To document breeding behavior, parentage was inferred by likelihood techniques when both parents were sampled and by parental genotype reconstruction when only one parent was sampled. In cases where neither parent was sampled, a sibship reconstruction approach was used. We found high mate fidelity and nest-site fidelity in urban peregrines; in 122 nesting attempts made by long-term breeders, only 12 (9.8%) mate changes and six (4.9%) nest-site changes occurred. Only one brood (of 35 tested) revealed extra-pair paternity and involved a male tending two offspring of a recently acquired mate. Natal dispersal patterns indicated that female peregrines dispersed on average 226 km, almost twice the distance of males (average 124 km). Despite the novel environment of cities, our results suggest that monogamous breeding, nest fidelity, and female natal dispersal are high in urban peregrines, not unlike other raptors living in non-urban habitats.

## Introduction

Urbanization and rapid expansion of the built environment are generally seen as threats to native species and biodiversity. But for populations of a few species, the built environment provides a novel and hospitable habitat. Such is the case for reestablished populations of peregrine falcons (*Falco peregrinus*) in the Midwestern United States. The effects of DDT during the 1950s and 60s caused the thinning of eggshells, contributing to low breeding success [1–3]. Peregrine falcons were considered extirpated east of the Great Plains by 1964 [4, 5]. However, the release of more than a thousand captive birds, many sired by parents of nonindigenous subspecies in the 1980s, successfully reintroduced peregrines to a new habitat in cities of the Midwest [5]. This population is now thriving in urban areas across 12 states (Minnesota, Wisconsin, Michigan, South Dakota, Nebraska, Iowa, Illinois, Indiana, Ohio, Kansas, Missouri, and Kentucky) and in portions of two Canadian provinces (the Lake Superior basin of Ontario, and southeastern Manitoba) [6]. Pre-DDT population levels have been exceeded in these areas, and their modern range includes regions where there were no historical breeding records (e.g., Ohio).

While peregrines in other regions also have rebounded, the Midwestern population is unique in being comprised almost entirely of urban-nesting birds. Cities benefit peregrine falcons by providing abundant prey, fewer predators/competitors, and plentiful nest sites. It has been reported that 8% of pairs nest on manmade substrates outside the Midwestern and Northeastern region [7] but urban nesting is very common east of the Mississippi River. Gahbauer et al. [8] report that of 152 nest sites identified in the Northeast, 87 (57.2%) were at urban sites. Urban nesting is even more ubiquitous in the Midwest; it was recently reported that over 91% of natal dispersal events were to urban nest sites [9]. In Illinois, peregrines historically nested on bluffs adjacent to the Mississippi River, but 90% of breeding pairs in the state currently nest on buildings and bridges in the Chicago area.

The urban environment undoubtedly influences the ecology, life history, and demography of species like peregrine falcons that have become established there. Studies are beginning to characterize several aspects of urban peregrine falcon biology, and because of the visibility and charisma of raptors, citizen participation has enhanced monitoring and data collection. For example, peregrine movements, density, and behavioral observations in the Midwest are annually reported and available in a public database (http://midwestperegrine.umn.edu/). Drawing on this database, Dennhardt and Wakamiya [9] report that natal dispersal distances for female peregrines averaged 226 km, more than twice that for males (108 km). Dispersal direction was skewed to the northwest and southeast towards urban centers and large bodies of water. In Northeastern North America, Gahbauer et al. [8] report that mortality was clearly linked to the built environment, with the most common cause of death for young birds being collisions with manmade objects including buildings, vehicles and power lines. For adult birds, however, territorial battles are common in urban populations [10, 11] and are the leading cause of mortality for adults in the Northeast [8]. In the Midwest from 1987 to 2009, there were reports of 46 territorial fights, and 16 of these fights involved the death of at least one member of a pair (http://midwestperegrine.umn.edu/). High breeding densities in cities may limit the urban peregrine’s ability to claim and keep their territories in subsequent seasons. Tordoff and Redig [10] suggest that the apparent durability of pair bonds in peregrines is attributable more to fidelity to territories than to mates, so higher territorial turnover could lead to higher mate turnover. Productivity of urban peregrines (average number of fledglings per nest) is among the highest documented in North America [12]. It is not uncommon for urban peregrines to have clutch sizes of 4–5 eggs [6, 13] while in natural habitats the peregrine typical clutch size is closer to three [14]. Once eggs hatch, fledgling success for urban peregrines appears to be closely related to the type of structure, nesting substrate, and whether nest trays or boxes are provided [8]. Furthermore, human assistance in rescuing fledglings that have come to the ground is quite common and likely increases fledgling success [8].

Extra-pair paternity (EPP) has not been studied in peregrine falcons, but in the only study to date in an urban raptor (and Accipitrid), Rosenfield et al. [15] report an extremely high frequency of EPP in the Milwaukee, Wisconsin population of Cooper’s hawks (*Accipiter cooperii*). They report that 19.3% of nestlings were extra-pair young (EPY), and 34% of all broods had at least one EPY. The authors suggest that females may accept extra-pair copulations (EPCs) in exchange for food, thus maximizing energy intake for egg production. They also suggest that recently formed high breeding densities in a food-rich urban area may contribute to this high rate of cuckoldry. For peregrine falcons in the Midwest, these and other factors might also lead to increased frequency of EPP. High fledging success may lead to increased competition for limited territories and attractive nest sites from non-territorial floaters. In addition, migrant birds, which breed in high latitudes, interact with resident birds as they pass through northward and may have the opportunity to mate [16]; urban centers in the Midwest (particularly in the Chicago area) lie on primary migration routes for arctic breeding peregrines. Finally, Rosenfield et al. [15] found that brood size was a positive predictor of EPP in nests in Milwaukee Cooper hawks, and Midwest peregrines typically have large clutch sizes, as mentioned above.

In this study we analyze the breeding and territorial behavior of Midwestern urban peregrine falcons. We achieve this by using a combination of field observations (geographical coordinates of nests, sightings of breeders, banding data from chicks, and reports of antagonistic behaviors), microsatellite genotyping, and multiple analytical approaches to infer kinship. Specifically, we assess nest site fidelity, mate fidelity, frequency of EPP, and natal dispersal, and then evaluate our findings in relationship to characteristics of the urban habitat (density and proximity of nests).

## Material and Methods

### Sampling

Since reintroduction programs started in the 1980’s, most peregrines born in the Midwestern region have been color-banded as chicks and blood samples taken at the time of banding. This usually occurs when nestlings are 21–35 days old. Nestlings can also be sexed at this time by weight and body measurements. Blood samples were collected by state peregrine monitors and stored at the Bell Museum of Natural History in Minneapolis, Minnesota, where blood samples (*n* = 349, Table A in S1 File) used for this study were on loan. In addition, one sample was on loan from the Field Museum of Natural History in Chicago, Illinois (Table A in S1 File). Samples included 282 chicks from 20 nest sites (Table 1) and 68 unrelated individuals: 29 breeders (Table 1) and 39 additional breeders and chicks from single-year nests (Table A in S1 File). Most of the samples (237/311, 76%) came from the Chicago area (IL) area with the rest coming from other Midwestern cities.

**Table 1:** Cities, nest sites, samples, and brood composition of Midwest peregrines analyzed in this study, including both genotyped and non-genotyped individuals. Samples were collected from 1997 to 2009. Values in parentheses indicate the number of genotyped individuals in each category. Unknown chicks were those observed but not sampled and sexed. Question marks indicate some uncertainty in the number of adults present at certain nests. A total of 28 breeders were genotyped and used for allele frequencies and summary statistics calculations.

	Adults		Chicks			
Nests	Female	Male	Female	Male	Unknown	Total
**Chicago area, IL**						
Broadway	2 (2)	3? (1)	23 (21)	16 (14)	4 (0)	
Evanston	2 (1)	3? (1)	13 (13)	7 (7)	1 (0)	
Hyde Park	1 (0)	1 (0)	8 (8)	5 (5)		
Pilsen	1 (0)	1 (0)	8 (8)	6 (6)		
Prison	3? (0)	3? (0)	10 (7)	11 (4)		
River	2 (1)	2 (0)	4 (4)	4 (3)	2 (0)	
Saint Michael	1 (1)	1 (1)	2 (2)	2 (1)	3 (0)	
UIC	1 (1)	2? (1)	12 (11)	12 (9)	2 (0)	
Uptown	2? (2)	2 (1)	8 (6)	16 (15)	3 (0)	
Wacker	4 (3)	4? (2)	24 (16)	26 (24)	2 (0)	
Waukegan	1 (1)	2? (0)	19 (16)	15 (14)	3 (0)	
South Loop	1 (0)	1 (0)	2 (2)	1 (1)		
Total	22 (12)	27 (8)	133 (114)	119 (103)	20 (0)	321 (237)
**Detroit, MI**						
Whittier	1 (1)	1 (0)	0	1 (1)		3 (2)
**Minneapolis-St. Paul-Monticello, MN**						
Colonnade	3 (1)	1? (0)	14 (11)	23 (20)		
Riverside	1 (1)	1 (0)	1 (1)	1 (1)		
NSP High Bridge	1 (1)	1 (0)	2 (2)	2 (2)		
NSP Monticello	1 (1)	1 (0)	4 (4)	0		
Total	6 (4)	4 (0)	21 (18)	26 (23)		57 (45)
**Cleveland, OH**						
Terminal Tower	2 (1)	1 (1)	3 (3)	5 (5)		11 (10)
**Jefferson-Milwaukee-Sheboygan, WI**						
Cargill Malt	1 (0)	1 (0)	4 (4)	1 (1)		
Froedtert Malt	1 (1)	1 (0)	0	1 (1)		
Landmark Lake	1 (0)	1 (0)	3 (3)	0		
WPL Edgewater	1 (1)	1 (0)	4 (4)	2 (2)		
Total	4 (2)	4 (0)	11 (11)	4 (4)		23 (17)

When blood samples from banded adults were not available, we considered such adults as “knowns,” because we know their origin (but not their genotypes) from their leg bands. Adults with no bands (or with bands that could not be unequivocally identified) were considered as “unknowns.” Also, not all chicks are banded (Table 1) because nests go undiscovered or are located in unreachable places, and nests located outside urban areas are monitored less frequently. Identity of banded birds, lifetime reproductive output, and nest locations were retrieved from an online database maintained by the Midwest Peregrine Society (http://midwestperegrine.umn.edu/).

#### Ethics Statement

No research protocol was needed for our study because samples were collected for general use by peregrine state monitors. The specimens used here are publicly deposited and accessible by others at the Bell Museum of Natural History in Minneapolis, Minnesota. In addition, one sample from Canada was on loan from the Field Museum of Natural History in Chicago. Samples for this study were on loan from these two permanent repositories.

### Microsatellite screening and genotyping

We used a modified enrichment protocol [17] to isolate microsatellites from two Midwestern peregrine falcons. Briefly, genomic DNA (gDNA) was digested with two restriction enzymes (RsaI and XmnI), and linkers (SuperSNX24) were used to ligate the ends of gDNA fragments. Biotinylated probes were hybridized to gDNA. Magnetic beads (Invitrogen) were added and a magnetic particle-collecting unit was used to capture the biotin-gDNA complex. Enriched fragments were amplified using PCR, cloned, and sequenced using the BigDye® Terminator v3.1 kit (Applied Biosystems) on an ABI3730 analyzer. Primers flanking core microsatellite repeats were developed using Primer3; http://frodo.wi.mit.edu/primer3/.

Genomic DNA was extracted from blood samples using the Qiagen DNeasy Tissue Kit^®^ following manufacturer’s protocol. Individuals were genotyped at a total of 11 loci: eight loci were developed as described above, and three loci (NVH *pf*13, NVH *fp*31 and NVH *fp*89) were developed by Nesje et al. [18]. PCR reactions in a total volume of 10 μl consisted of 1XPCR Buffer (10 mM Tris-HCl, 1.5 mM MgCl_2_, 50 mM KCl, pH 8.3), 0.6 μM of each primer, 200 μM each dNTP, 0.6 U *Taq* and approximately 1 μl of 10ng/μl gDNA. Thermal cycling was as follows: 94°C for 4 min, 35 cycles at 94°C for 30 s, *T*_a_° for 20 s) and 72°C for 15 s. For fragment analyses, 1 μl of PCR product was mixed with 9 μl of a solution of GeneScan^TM^ 500 LIZ^TM^ Size Standard (Applied Biosystems) and formamide, and run on an ABI3730 DNA Analyzer. Genotyping was carried out using the STRand Analysis Software v.2.4 [19].

### Kinship, breeding and natal dispersal analyses

For evaluating our microsatellite genotypes, deviations from Hardy-Weinberg equilibrium, and the presence of null alleles were tested in CERVUS v.3.0 [20, 21], while linkage equilibrium was tested in GENEPOP on the web [22, 23]. Population allele frequencies needed in parentage assessment and population summary statistics were carried out with a sample of 68 unrelated birds: 29 breeders (Table 1) and 39 breeders and chicks (Table A in S1 File) genotyped at 11 microsatellite loci (Table 2).

**Table 2:** Repeat motifs, primers sequences, sizes, and GenBank Accession numbers for 11 peregrine falcons microsatellite markers. The CAB markers were developed specifically for this project while the NVH markers are from Nesje et al. [18].

		Primers	(5' → 3')			GenBank
Locus	Repeat motif	Forward (label)	Reverse	*T*_a_°	Size range	Accession no.
CAB *fp*18-2	[AGTTAAGTT]_28_	ACAGGATACATGCTTTGTAGCTC (FAM)	GCCACCAGGACCAAATTTCT	54	158–482	JQ914586
CAB *fp*24	[TATTC]_17_[TGTCC]_6_	AACCCCCATGATGAACAAGA (NED)	CATTGCAAACATCTCCATAGTCA	54	211–286	JQ914587
CAB *fp*77	[TGGA]_6_GA[TGGA]GA[TGGA]_8_	TCTTCCATCTGGGCTTCATT (NED)	ATCCTCCTGCCAAAGCAACT	51	241–281	JQ040496
CAB *fp*85	[TATC]_10_	TGCCAGTCAGGTCACAATTT (VIC)	CCCCACGGAAATTAATAGACTT	55	238–258	JQ914588
CAB *fp*117	[CATA]_7_ CATG [CATA]_4_	TGTGCCTACCCAAAAGCAAT (PET)	ACATCCAAAAGTGGCACCTC	56	246–270	JQ914589
CAB *fp*120	[TATC]_15_	TGGTAGGCATTTGGATGTGA (NED)	CGCAGGTTTCTTGTGCTGTA	55	261–277	JQ914590
CAB *fp*157	[TCTA]_14_	GAGGGGAAAAATTGTGGGATA (FAM)	TTGGAAAGCATATTGCATCG	55	200–236	JQ914591
CAB *fp*181	[TCTTA]_11_	CCTGAAAACCTGTCATGTCCT (PET)	TCAGGCCCCTTTGAGATTAAGA	57	178–368	JQ914592
NVH *fp*13	[CA]_12_	AGCTTGATTGAGGCTGTG (VIC)	CCAAATTCCCTGCTGAAG	61	115–131	AF118421
NVH *fp*31	[CA]_17_	ATCACCTGCACATAGCTG (NED)	TTTAGCTCCTCTCTCTCAC	51	165–177	AF118422
NVH *fp*89	[AT]_12_	CTCTGCCCTGAATACTTAC (FAM)	GAATCTTGTTTGCATTGGAG	58	142–162	AF118430

**Fig 1:**
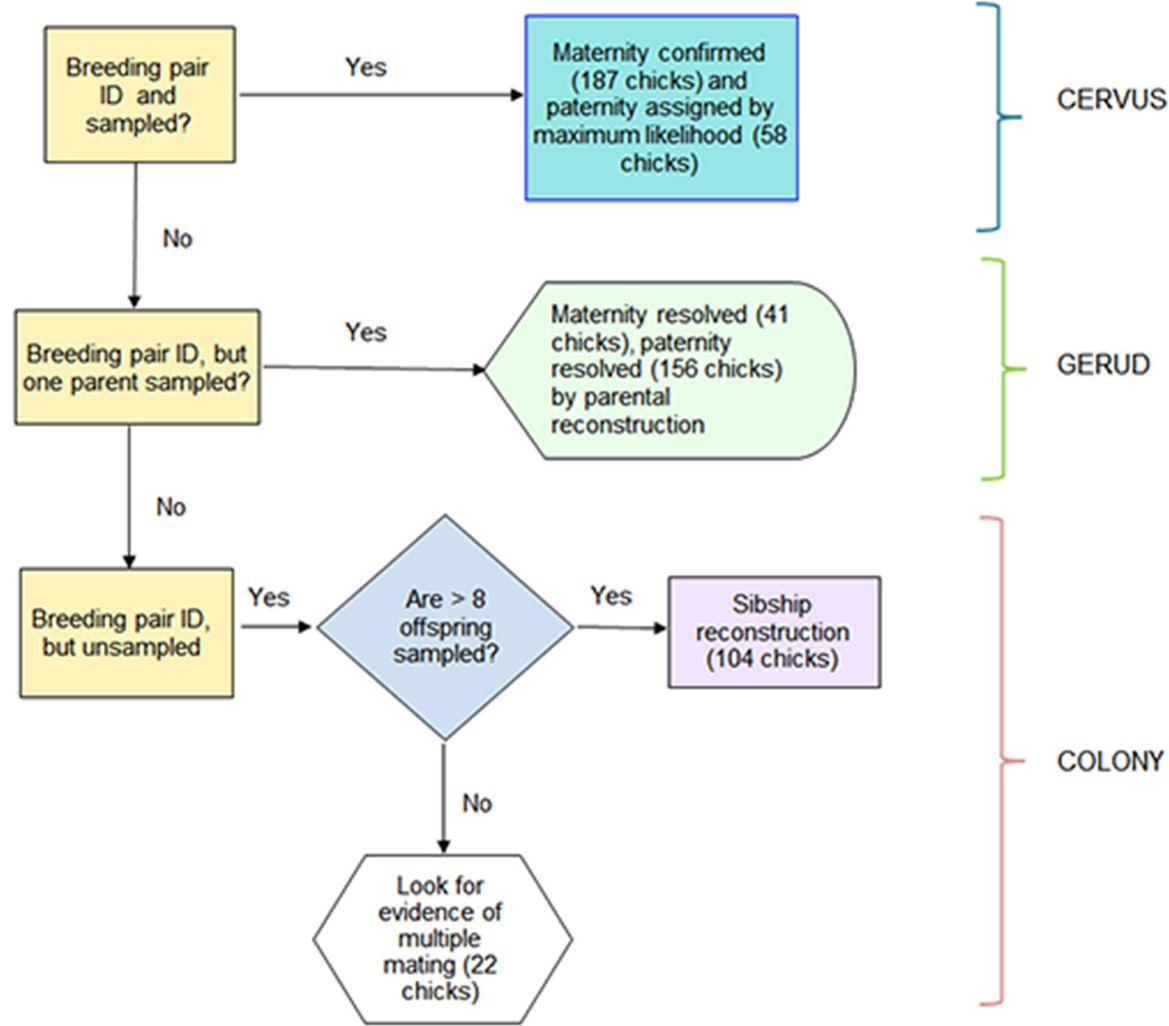
Work flow illustrating the sequence of analyses and software used in this study, and the number of peregrine chicks assigned to parents by each type of analysis. ID means that adult was banded as a chick.

Banding and genetic data were incorporated in the analyses of parentage, sibships and ultimately breeding behavior using a workflow (Fig 1) with parentage initially assigned using the likelihood-based approach implemented in CERVUS. This method considers the proportion of candidate parents sampled, allows for genotyping errors (null alleles, mistypes), and calculates statistical confidence based on the difference in LOD scores of candidate parents, Delta Δ, (i.e. the natural log of the likelihood ratio). CERVUS estimates statistical confidence in the assignment of parentage using a series of parallel simulations. The simulations were done with and without maternal genotypes on a subset of chicks (*n* = 191). Simulation parameters values were calculated from our dataset (values in parentheses): 1- number of candidate males estimated from field data (*n* = 25), 2- proportion of candidate males sampled (0.641); 3- proportion of loci typed, averaged across all loci and individuals (0.991), and 4- error rate, which accounts for the fraction of loci typed incorrectly averaged across all loci and individuals (0.022). The final phase of the simulation finds critical values of Δ for relaxed (80%) and strict (95%) confidence levels based on 10,000 permutations. Candidate fathers were any tending males found in a given nest and in nearby nests. Mother-father pairs were allocated parentage if they were assigned at 95% or higher confidence. Males were allocated parentage with at least 80% confidence and if there were zero or one parent-offspring mismatches. Maternity was confirmed by exclusion using cervus for all cases when the maternal genotype was available. Mismatches were interpreted as genotyping errors.

In cases where the paternal sample was not available, the paternal genotype was inferred through paternity reconstruction using gerud 2.0 [24]. This program uses an exhaustive algorithm to reconstruct the minimum set of parents that can explain the progeny array [24] which are assumed to be composed of full or half sibs. To assess how frequently gerud recovers the correct number of parents, as well the correct paternal and maternal genotypes under the assumed pattern of parentage, we ran simulations with gerudsim2.0. Progeny arrays are simulated based on population allele frequencies. Generally, the best results are obtained using 2–4 loci that have the highest exclusion probabilities [25]. Finally, in cases where neither parent was sampled, a sibship reconstruction approach was used to calculate the most likely number of parents contributing to a progeny array by inferring sibling relationships among offspring, using colony [26].

Based on kinship analysis that identified breeders and matched them to their nests, we used the data from breeders from nests with long-term data (more than 6 years of continuous data). These nests were in the Chicago area (Broadway, Evanston, UIC, Uptown, Wacker, and Waukegan, Table 3) and were used to measure nest-site and mate fidelity. We defined nest site fidelity as returning to breed at the exact same site or in close proximity, i.e. <20m, or same structure but different ledge. We then performed a generalized mixed effects model (GLMM) to assess variation in nest site fidelity. Fixed effects included biological relevant covariates characterizing breeders (sex and previous breeding success) and their spatial relationship with other breeders (distance to the two closest nests and the total number of occupied nests in the Chicago area in that year). We added bird identity as a random effect to account for pseudo-replication, (i.e. the same bird being measured more than once). Because nest site fidelity is a binary response variable (birds return or do not return to breed), we fitted the nest site fidelity model with a binomial error structure to the data. Statistical analyses were done with R v.3.1.2 [27] using the package *lme4* [28, 29].

**Table 3:** Reproductive output, nest site fidelity and mate fidelity from six nest sites (Chicago, IL) with long-term observation data confirmed by genetic analyses. Numbers in parentheses indicate offspring not available for genotyping.

Male ID, Nest site	Lifetime	Years	Number of	Number
	Production	nesting	Nest sites	of Mates
Hubert, Wacker	26 (+9)	12	1	4
Unbanded 1, Waukegan	18 (+4)	5	1	1
P/M, Broadway	18 (+1)	5	2	2
G/G, Uptown	16 (+6)	6	1	1
95T, Broadway	12 (+6)	10	2	1
0/*A, UIC	12 (+5)	6	2	1
48/M, Evanston	12	3	1	1
Unbanded 2, Waukegan	12	2	1	1
59/H, Wacker	6	2	1	1
Joel, Evanston	5	1	1	2
Unbanded 1, UIC	3	3	1	1
Unbanded 2, UIC	5	1	1	1
Unbanded, Uptown	5	2	1	1
7/6, Wacker	6	2	1	1
91/E, Wacker	0	1	1	1
Female ID, Nest site				
5/*X, Waukegan	30 (+7)	7	1	2
*4/H, Uptown	24 (+7)	8	2	2
5/*P, Broadway	23	5	1	1
*6/D, UIC	21 (+6)	10	1	3
64/D, Evanston	18	4	1	2
*C/4, Wacker	15 (+5)	5	1	1
01/A, Wacker	13	3	2	2
2/8, Broadway	12 (+6)	10	1	1
*P/*5, Wacker	11 (+5)	5	2	2
1/*D, Evanston	2	1	1	1
E/E, Wacker	2	1	1	1
Unknown 1, Wacker	0	1	1	2
Unknown 2, Wacker	0	1	1	1

To study natal dispersal, we identified birds from the peregrine database (http://midwestperegrine.umn.edu/) where data on hacking or natal site and first breeding site were available. Using the location of nests (latitude and longitude), we determined natal dispersal distance, as defined by the straight-line distance from hack or natal site to the first breeding site.

## Results

Our set of microsatellites (Table 2) proved suitable for carrying out parentage and sibship analyses. Birds were genotyped at 99% of loci. The mean number of alleles per locus was 8.64 and the mean gene diversity was 0.73. No linkage disequilibrium was detected. Probability of identity among siblings (PID sib) was 5.299 x 10^−5^ (i.e., 0.005% of full siblings share the same genotype by chance) and no individuals had the same genotype. All loci had 5–13 alleles per locus (*k*), high observed heterozygosity (*H*_O_ = 0.516–0.871) and expected heterozygosity (*H*_E_) values above 0.5, making them optimal for parentage analyses [21] (Table 4). Three loci were not in Hardy-Weinberg equilibrium after applying a Bonferroni correction for multiple comparisons (*P*<0.05) [30]. One locus (CAB *fp*181) exhibited heterozygote deficit possibly due to null alleles. Two other loci (CAB *fp*24, CAB *fp*157) exhibited an excess of heterozygotes. These deviations should not be problematic for parentage analysis (*see* cervus documentation). The combined non-exclusion probability from simulations in CERVUS was 6.2 10^−7^, which represents the probability of falsely excluding the true parent pair of a given offspring if both parents were sampled. To assess how accurately gerud could recover the correct number of parents with our markers, as well as the correct paternal and maternal genotypes, we ran simulations with gerudsim2.0. The parental reconstruction and determination of the number of sires depend on the sample size and population allele frequencies. Results of the simulation indicated that when combined broods included 5–30 chicks from consecutive years, the probability of inferring the correct number of sires was 90% or higher, but lower for smaller brood sizes. In nests where blood samples of males were unavailable, the proportion of correct reconstructed paternal genotypes was 70% or higher. These simulations support the robustness of results given by gerud on the reconstructed parental genotypes.

**Table 4:** Summary statistics for 11 microsatellite loci from 350 genotyped peregrine falcons. Number of alleles (*k*), observed heterozygosity (*H*_O_), expected heterozygosity (*H*_E_), Hardy Weinberg (HW).

Locus	*k*	*H*_O_	*H*_E_	HW
CAB *fp*18-2	11	0.516	0.522	NS
CAB *fp*24	12	0.871	0.832	*
CAB *fp*77	10	0.763	0.783	NS
CAB *fp*85	5	0.575	0.578	NS
CAB *fp*117	9	0.746	0.779	NS
CAB *fp*120	5	0.594	0.651	NS
CAB *fp*157	9	0.804	0.769	***
CAB *fp*181	13	0.699	0.82	**
NVH *fp*13	8	0.836	0.806	NS
NVH *fp*31	6	0.764	0.741	NS
NVH *fp*89	7	0.777	0.777	NS

### Parentage and sibship assignments

A challenge in parentage analysis is the sampling of candidate parents. Because not all the breeders were genetically sampled, we needed three different approaches to infer familial relationships (Fig 1). Parentage and sibship assignments were attempted for a total of 282 offspring from 149 broods, and these broods ranged from one to five chicks in size. We started with maternity assignments using cervus on 212 out of 282 offspring (75%) from 20 nests (Table 5). Maternity was assigned to 191 (90%) of these offspring. These included 186 assignments where the genotype of the female tending the nest was known and there were no mismatches between mother and offspring, and five assignments where mother-offspring mismatches occurred at one locus in five chicks from four nests. In these cases, maternity assignments were confirmed by mother-father-offspring trios assigned at 95% confidence. Maternity was unresolved for 17 chicks in the Chicago area and four chicks in Cleveland because no genetic data from the adult female was available.

**Table 5:** Parentage assignments for urban peregrine falcon chicks in the Midwest.

		Maternities resolved				Paternities resolved				
Nest	No. of chicks^a^	Exclusion	Parental reconstruction	Sibship reconstruction	No. of mothers^b^	Categorical allocation		Parental reconstruction	Sibship reconstruction	No. of fathers^b^
						80%	95%			
**Chicago area,**	**IL**									
Broadway	35	12+23*	–	–	2	2	16	12+5*	–	3
Evanston	20	2	18	–	2	–	5	3+8	4	3
Hyde Park	13	–	–	13	1	–	–	–	13	2
Pilsen	14	–	–	14	1	–	–	–	14	1
Prison	11	–	–	3+8*	2	–	–	–	5+6*	2
River	7	1	–	6	2	–	–	–	6+(1)^∫^	2
Saint Michael	3	3	–	–	1	2	1	–	–	1
UIC	20	20	–	–	1	–	12	3+5*	–	3
Uptown	21	21	–	–	1^c^	3	13	5	–	2
Wacker	40	15+11+12 +2*	–	–	4	–	6	26+2+6*	–	4
Waukegan	30	30	–	–	1	–	–	18+12*	–	2
South Loop	3	–	–	3	1	–	–	–	3	1
**Detroit, MI**										
Whittier	1	1	–	–	1	–	–	–	(1)^∫^	
**Minneapolis-**	**St. Paul-**	**Monticello,**	**MN **							
Colonnade	31	12	12+7*	–	3^d^	–	–	31	–	1
Riverside	2	2	–	–	1	–	–	–	(2)^∫^	
NSP High Bridge	4	4	–	–	1	–	–	4	–	1
NSP Monticello	4	4	–	–	1	–	–	4	–	1
**Cleveland,**	**OH **									
Terminal Tower	8	4	4	–	2	–	8	–	–	1
**Jefferson-**	**Milwaukee-**	**Sheboygan,**	**WI **							
Cargill Malt	5	5	–	–	1	–	–	5	–	1
Froedtert Malt	1	1	–	–	1	–	–	–	(1)^∫^	
Landmark Lake	3	–	–	3	1	–	–	–	3	1
WPL Edgewater	6	6	–	–	1	–	–	4+2*	–	2
**TOTAL**	282	191	41	50	32	7	61	155	59	34

^a^ This accounts for chicks genotyped only

^b^ Number of mothers (or fathers) determined after analysis

*Each number represents size of full-sib group, and each sib-group is separated by the symbol +

^∫^Offspring size not enough to resolve paternity

^c^ Number of mothers reported (2) differed from results after genetic analysis

^d^ Number of mothers reported (4) differed from results after genetic analysis

Paternity assignments using cervus were done on 68 of the 191 chicks with assigned maternity, 61 at the 95% confidence level and seven at the 80% confidence level (Table 5). In addition, we reconstructed the genotype of unsampled parents with gerud for 167 chicks. From this last group, 34 chicks had both parental genotypes reconstructed, 126 chicks had only their father’s genotypes reconstructed and seven chicks had only their mother’s genotypes reconstructed. The father’s genotypes could not be reconstructed for five remaining chicks from four broods. In these cases, paternal alleles could not be established without ambiguity.

Sibship reconstruction using colony resulted in nine full-sibs groups from 11 nest sites; results that were in agreement with banding data (Table 5). For one nest (Prison, Chicago area), where 11 chicks were sampled, the sibship reconstruction identified three full-sib groups. Here, one full-sib group with six chicks was in agreement with banding data but parents were unsampled. The remaining five chicks consisted of two sibgroups and they had unknown parents. In these cases, even without banding data and/or unsampled parents, we could infer full sib groups and therefore whether parents were the same throughout the years. However, chicks from four broods (Chicago area, Detroit, Minneapolis, and Milwaukee, Table 5) could not have the paternal genotype reconstructed (even though maternity was resolved) because each brood had one or two chicks.

### Fidelity to nest sites and mates, EPFs and natal dispersal

Nest site fidelity and mate fidelity were assessed for the nests with long-term data (more than 6 years of continuous data) in the Chicago area (Broadway, Evanston, UIC, Uptown, Wacker, and Waukegan, Table 3). From a total of 122 nesting attempts, only six (4.9%) involved changes to a new nesting site. Two of these involved males moving from other states to two new territories in the Chicago area. One was a female that bred in Chicago, then was injured but after recovering went to breed in another state. Three changes were to nearby nest sites that occurred after previous breeding attempts had been unsuccessful or a mate disappeared. For the same group of 122 nesting attempts, 12 mate changes occurred (9.8%). Six involved changes after a mate died or presumably died, three involved disappearance of mates following territorial fights, and three involved mate changes in association with changing nest sites.

Nest site fidelity was positively correlated with previous breeding success (GLMM: *z* = 2.094, *P* = 0.0365). Breeders that were unsuccessful were more likely to nest in a different site the following season (40%) than successful breeders (10%). There were no differences between sexes (GLMM: *z* = -0.573, *P* = 0.581). Local breeding density in the peregrine urban habitat did not play a role in nest site fidelity; neither distance to the two nearest neighbor nests (GLMM: *z* = 0.917, *P* = 0.3589) nor the total number of active nests (GLMM: *z* = -0.958, *P* = 0.367) were correlated with nest site fidelity.

To test for EPP, we examined 35 broods with 126 chicks from four nests in the Chicago area (Broadway, Uptown, Wacker and Waukegan). These nests were chosen because we had observational and genetic data from candidate fathers (other than the male tending the nest). Parental reconstruction indicated that two nestlings out of 40 were not fathered by the male tending the nest (Wacker, Chicago). This male paired with a single female from 1993 to 1997. In 1998, however, this female disappeared and the tending male paired with a new female. Thus the two chicks born that year to his new mate were not his. If this is considered an EPP event, the studied Chicago area nests had an EPP rate of two of 126 (1.58%) young and one of 35 (2.85%) broods.

Lastly, natal dispersal showed large individual variation with birds of both sexes (25 males, 29 females) moving short distances (≤ 100 km, 33%) or even settling back at the hack or natal site (4%), while others moved longer distances (>100 km, 63%). Females peregrines moved on average almost twice as far as males, 226 km vs. 124 km (χ^2^ = 5.649, df = 1, *P* < 0.05).

## Discussion

Our study contributes new information on a recovered top avian predator, one that is adapting to a novel urban environment. Using genotype data from 350 peregrine falcons sampled in urban areas in the Midwest, and using a combination of parentage assignment and reconstructed parental genotypes, we resolved the maternity of 265 of 282 (94%) offspring of banded female breeders and resolved the paternity of 201 of 282 (71%) offspring of banded male breeders. We also reconstructed genotypes for an additional 18 of 37 male breeders (48%), 12 unbanded and six banded but unsampled. Our study highlights the value of genetic data from offspring in cases where samples were not available; through analysis of offspring genotypes, we were able to reconstruct genotypes of unknown or unsampled breeders for our analysis (Table B in S1 File). Combining this new information on kinship and genotypes with long-term field data allowed us to characterize several aspects of urban peregrine breeding behavior and dispersal.

We found that Midwest urban peregrines show high fidelity to both mates and nest sites. While these behaviors are characteristics of raptors, they have not been studied extensively in populations living in urban environments. Higher breeding densities [31], higher productivity [13], frequent territorial battles [8, 11], and variability in nesting structures [8] are among many factors that might lead to differences in breeding behaviors in urban populations. In the 12 cases of mate changes, nine (60%) involved the presumed death of the mate. Nest site fidelity was even higher than mate fidelity, with only six observed changes in nest sites. Thus, our data shows that mate turnover is higher than territorial turnover, supporting Tordoff and Redig [10] hypothesis regarding apparent durability of pair bonds. Nest sites are more ‘stable’ entities to which the birds are attracted compared to their mates (which may or may not be there to breed in subsequent years). There was no significant difference between males and females in nest site fidelity. Unsuccessful breeders were more likely to switch nest sites than individuals that bred successfully. Given that structure type and substrate has been shown to be correlated with productivity [8], moving to a different nearby structure could increase chances for successful reproduction. Indeed, five of the six moves were to nearby nest sites. While we did not include structure type or substrate in our analysis, Gahbauer et al. [8] report that productivity was higher for peregrines nesting on buildings where nests had overhead cover. Also, direct human assistant by peregrine monitors in returning grounded fledglings to nests may artificially increase reproductive success and nest site fidelity. Meanwhile, there was no significant correlation between nest site fidelity and breeding density. This suggests that other aspects of nest sites may be more important in breeding success than interference from other breeders for urban peregrines. The high return rate to previously successful nest sites may have been a key factor in peregrines’ rapid recovery, with productive sites being continually occupied by productive pairs. Also, when an established breeder at a productive nest site fails to return it is quickly replaced by another bird. Our finding of very high nest site fidelity is in agreement with reports of other raptor species [32] with a few noteworthy exceptions. Peregrine falcons in northern Spain switched nest sites on average every three years, and were more likely to change nest sites after raising a large brood [33]. Gyrfalcons are faithful to territories but display low nest site fidelity with no dependence on previous breeding success (22%, [34]). Rotating among alternate sites within a territory is also common in American kestrels [35]. Accipiters [35] and eagles [36] also move nest sites. It would therefore be interesting to compare nest site fidelity in urban peregrines to that of peregrines nesting in more traditional habitats, but to our knowledge the latter has not been investigated.

As mentioned in the introduction, a very high frequency of EPP was recently reported for an urban Midwestern population of Cooper’s hawks (*A*. *cooperii*, [15]). Brood size was a good predictor of EPP in this population. Mougeot [31] found that EPC frequency in raptors was positively correlated with breeding density. These findings suggested that we might find relatively high rates of EPP in urban peregrines. However, we found only two offspring from one nest that could be considered the product of EPP, yielding a rate of only 1.58%. Further, there were special circumstances associated with this EPP. The tending male lost his mate in 1999 and remated with a female likely already impregnated by another male (Table 5). Genetic data confirmed that this male nested from 1993 until 2004 and sired 26 chicks over this time. Thus, “widowed” males tending a nest may, in such cases, accept new females already impregnated by another male because of long-term reproductive benefits. Although our paternity analysis included males from neighboring nests, we did not find the sire of the EPP chicks, so it is possible that the male involved in the EPP event was a floater. The only other reported case of EPP in peregrines also was in an urban nest and there were also special circumstances [37]. An established breeder who lost its territory after a fight established a new territory, and DNA fingerprinting indicated that this male fathered chicks in both the old nest and the new nest. Our finding of a very low rate of EPP agrees with reports in other falcons nesting in natural habitats. While Falconids have been shown to be more closely related to Passerines than they are to other raptors [38], they have lower rates of EPP compared to passerine birds. Low rates of EPP (from zero to ~7%) have been reported in merlins (*F*. *columbarius*, [39]), lesser kestrels (*F*. *naumanni*, [40, 41]), and American kestrels (*F*. *sparverius*, [42]).

Low EPP in urban peregrines despite increasing breeding density may be explained by parental care and mechanisms of paternity assurance. In long-lived species like peregrines and other Falconids in which bi-parental investment is substantial, females may avoid extra-pair copulations because males reduce their breeding effort when their paternity is in doubt [43]. Our results support the hypothesis that in places where competition for nests and mates is high, sperm competition will also be high [31]. Low EPFs may be the result of behaviors aimed to ensure paternity such as displays that enhance pair bonding and a high frequency of within-pair copulations. Peregrines and most other monogamous raptors perform conspicuous copulation displays [44, 45] and a great majority copulate up to several hundred times a clutch during the fertile period (~ three weeks) [31]. Given this level of interaction among successful pairs, extra-pair copulations may be costly to pursue in terms of time and risk of injury or death due to interactions with the territorial male.

Our findings on natal dispersal distances are in general agreement with those reported by Dennhardt and Wakamiya [9] and previous studies on the Midwest population [10]. We looked at a subset of the data Dennhardt and Wakamiya [9] used and obtained similar estimates of dispersal distance. We found the average dispersal distances of females in both studies were 226 km, about twice that for males (124 km here and 108 km in [9]). Urban-recovered peregrine populations from the Eastern US [46] and from Northern Spain [47] also show female-biased dispersal. While there has been variation among studies in estimated dispersal distances for both sexes, the general trend of females travelling further than males is universal. Some individuals (usually males) settle at their hack or natal site while others (usually females) move hundreds of km to different (urban) areas. The concentration of nest sites or food resources in urban areas may influence variation in dispersal distances. Many individuals may disperse shorter distances, away from natal sites but in the same urban center. Dispersal distances for those that disperse to new urban centers will depend on the proximity of suitable urban habitats [9].

Urban peregrine falcons offer an important system for continued study, not only because of their remarkable recovery, but also because their populations will continue to adapt to anthropogenic landscapes. Peregrine falcons have become highly visible icons of urban wildlife that promote public education and citizen science. Our study was greatly enhanced by combining molecular markers with field observations. Blood samples of banded chicks are being collected every year, and long term field monitoring has produced an extensive, accessible database. Therefore the opportunity exists to continue our approach and to monitor and study the ecology of urban peregrine falcons.

## Supporting Information

S1 File **S1 File contains: Table A in S1 File. List of all specimens used in the study with their US Fish and Wildlife identification number (USFWS#), birth year, sex, and locality information from the Bell Museum of Natural History in Minneapolis.** Additional samples (*n* = 39) not shown in Table 1 used in the study to calculate population allele frequencies and summary statistics are denoted with an asterisk. Note: Only one sample (from Canada) denoted with ^1^ was on loan from the Field Museum of Natural History in Chicago. **Table B in S1 File. Detailed contribution from field data and genetic data on the kinship relationships for all Midwestern surveyed nests.**
(DOCX)
