# Supplementary material for: Sex in the City: Breeding Behavior of Urban Peregrine Falcons in the Midwestern US
Source: PLoS One. 2016 Jul 15;11(7):e0159054. doi: 10.1371/journal.pone.0159054 (PMC4946791; doi:10.1371/journal.pone.0159054)
Supplement: S1 File — contains: Table A in S1 File. List of all specimens used in the study with their US Fish and Wildlife identification number (USFWS#), birth year, sex, and locality information from the Bell Museum of Natural History in Minneapolis. Additional samples (n = 39) not shown in Table 1 used in the study to calculate population allele frequencies and summary statistics are denoted with an asterisk. Note: Only one sample (from Canada) denoted with 1 was on loan from the Field Museum of Natural History in Chicago. Table B in S1 File. Detailed contribution from field data and genetic data on the kinship relationships for all Midwestern surveyed nests. (DOCX) [file pone.0159054.s001.docx]

Supplementary Tables (S1 File)

**Sex in the city: Breeding behavior of urban peregrine falcons in the Midwestern US**

I. C. Caballero*, J. M. Bates, M. Hennen, M. V. Ashley

*Correspondence: I. C. Caballero Department of Biology, Texas A&M University, 3258 TAMU, College Station, TX 77843.

E-mail: [icabal@tamu.edu](mailto:icabal@tamu.edu)

Tel: (979)- 845-0925

Fax: (979)-845-2891

Table A in S1 File. List of all specimens used in the study with their US Fish and Wildlife identification number (USFWS#), birth year, sex, and locality information from the Bell Museum of Natural History in Minneapolis. Additional samples (n = 39) not shown in Table 1 used to calculate population allele frequencies and summary statistics are denoted with an asterisk. Note: One sample (from Canada) denoted with ^1^ was on loan from the Field Museum of Natural History in Chicago.

| USFWS# | Year | Sex | Site | State |
| --- | --- | --- | --- | --- |
| R0027351 | NA | male | NA | Minnesota |
| R0027388 | NA | female | NA | Minnesota |
| 877-42484 | 1985 | female | Castle Cliff | Minnesota |
| 987-20710 | 1985 | male | Castle Cliff | Minnesota |
| 816-21947 | 1988 | male | Mayo Clinic | Minnesota |
| 816-22000 | 1989 | male | North Central Life | Minnesota |
| 2206-13814* | 1990 | male | Glenn Ellyn | Illinois |
| 2206-13821 | 1990 | male | Cedar Rapids | Iowa |
| 1807-29475 | 1991 | female | Cincinnati | Ohio |
| 1807-34844 | 1992 | female | Woodmen Tower | Nebraska |
| 1807-34872 | 1993 | female | WPL Edgewater | Wisconsin |
| 1807-34888 | 1993 | female | Wacker | Illinois |
| 1807-34889 | 1993 | female | Wacker | Illinois |
| 2206-18503 | 1993 | male | WPL Edgewater | Wisconsin |
| 2206-18504* | 1993 | male | Akron | Ohio |
| 2206-18519 | 1993 | male | Wacker | Illinois |
| 2206-18526 | 1993 | male | Colonnade | Minnesota |
| 2206-18527 | 1993 | male | Colonnade | Minnesota |
| 2206-18528 | 1993 | male | Colonnade | Minnesota |
| 1807-49762 | 1994 | female | Colonnade | Minnesota |
| 1807-49764 | 1994 | female | Colonnade | Minnesota |
| 1807-49765 | 1994 | female | Wacker | Illinois |
| 1807-49766 | 1994 | female | Hyde Park | Illinois |
| 1807-49767 | 1994 | female | Broadway | Illinois |
| 1807-49768 | 1994 | female | Broadway | Illinois |
| 1807-49776 | 1994 | male | Colonnade | Minnesota |
| 1807-49789 | 1994 | female | Woodmen Tower | Nebraska |
| 2206-25403 | 1994 | male | Wacker | Illinois |
| 2206-25404 | 1994 | male | Wacker | Illinois |
| 2206-25408 | 1994 | male | Colonnade | Minnesota |
| 1807-53833 | 1995 | female | Broadway | Illinois |
| 1807-53834 | 1995 | female | Broadway | Illinois |
| 1807-53835 | 1995 | female | Wacker | Illinois |
| 1807-53836 | 1995 | female | Hyde Park | Illinois |
| 1807-53837 | 1995 | female | Hyde Park | Illinois |
| 1807-53850 | 1995 | male | Colonnade | Minnesota |
| 2206-25464 | 1995 | male | Colonnade | Minnesota |
| 2206-25465 | 1995 | male | Colonnade | Minnesota |
| 2206-25466 | 1995 | male | Broadway | Illinois |
| 2206-25467 | 1995 | male | Wacker | Illinois |
| 2206-25468 | 1995 | male | Wacker | Illinois |
| 2206-25469 | 1995 | male | Colonnade | Minnesota |
| 1807-53861 | 1996 | female | Bailly Power Plant | Indiana |
| 1807-53956 | 1996 | female | Froedtert Malt | Wisconsin |
| 1807-53961 | 1996 | female | NSP Monticello | Minnesota |
| 1807-53969 | 1996 | female | Colonnade | Minnesota |
| 1807-53974 | 1996 | female | Broadway | Illinois |
| 1807-77765* | 1996 | female | Froedtert Malt | Wisconsin |
| 2206-35709 | 1996 | male | Colonnade | Minnesota |
| 2206-35861 | 1996 | male | Rhodes State Office Tower | Ohio |
| 2206-35892 | 1996 | male | Colonnade | Minnesota |
| 2206-35893 | 1996 | male | Colonnade | Minnesota |
| 1807-53883 | 1997 | female | Bong Bridge | Minnesota |
| 1807-53976 | 1997 | female | Wacker | Illinois |
| 1807-53977 | 1997 | female | Broadway | Illinois |
| 1807-53978 | 1997 | male | Broadway | Illinois |
| 1807-53979 | 1997 | female | Broadway | Illinois |
| 1807-53987 | 1997 | female | Hyde Park | Illinois |
| 1807-61902* | 1997 | female | Book Building | Michigan |
| 1807-61906 | 1997 | male | Whittier Apts | Michigan |
| 1807-61912 | 1997 | female | NSP Monticello | Minnesota |
| 1807-61913 | 1997 | female | NSP Monticello | Minnesota |
| 1807-61914 | 1997 | female | NSP Monticello | Minnesota |
| 1807-61928 | 1997 | female | City Center | Minnesota |
| 1807-61930 | 1997 | female | North Central Life | Minnesota |
| 1807-61932 | 1997 | female | Mendota Bridge | Minnesota |
| 2206-35871 | 1997 | male | Wacker | Illinois |
| 816-66334* | 1997 | male | Monsanto Creve | Missouri |
| 1807-61922 | 1998 | female | River | Illinois |
| 1807-61924 | 1998 | female | Broadway | Illinois |
| 1807-61925 | 1998 | female | Broadway | Illinois |
| 1807-61926 | 1998 | female | Hyde Park | Illinois |
| 1807-61937 | 1998 | female | Prison | Illinois |
| 1807-61938 | 1998 | female | Prison | Illinois |
| 1807-61974 | 1998 | female | Riverside Plant | Minnesota |
| 1807-69754 | 1998 | female | Colonnade | Minnesota |
| 2206-35708 | 1998 | male | Broadway | Illinois |
| 2206-35732 | 1998 | male | Hyde Park | Illinois |
| 2206-35733 | 1998 | male | Hyde Park | Illinois |
| 2206-35734 | 1998 | male | Wacker | Illinois |
| 2206-35735 | 1998 | male | Wacker | Illinois |
| 2206-35768* | 1998 | male | Sherco Plant Becker | Minnesota |
| 2206-35770 | 1998 | male | Riverside Plant | Minnesota |
| 2206-41001 | 1998 | male | Prison | Illinois |
| 2206-41013 | 1998 | male | Colonnade | Minnesota |
| 2206-41014 | 1998 | male | Colonnade | Minnesota |
| 2206-41015 | 1998 | male | Colonnade | Minnesota |
| 1807-44144 | 1999 | female | Pittsburgh | Pennsylvania |
| 1807-61982 | 1999 | female | Colonnade | Minnesota |
| 1807-69793 | 1999 | female | NSP High Bridge | Minnesota |
| 1807-69794 | 1999 | female | NSP High Bridge | Minnesota |
| 1807-77601 | 1999 | female | Hyde Park | Illinois |
| 1807-77602 | 1999 | female | UIC | Illinois |
| 1807-77603 | 1999 | female | UIC | Illinois |
| 1807-77617 | 1999 | female | Landmark on the Lake | Wisconsin |
| 1807-77625 | 1999 | female | High Bridge | Wisconsin |
| 1807-77766 | 1999 | female | WPL Edgewater | Wisconsin |
| 2206-41055 | 1999 | male | UIC | Illinois |
| 2206-41056 | 1999 | male | Prison | Illinois |
| 2206-41081 | 1999 | male | NSP High Bridge | Minnesota |
| 2206-41082 | 1999 | male | NSP High Bridge | Minnesota |
| 1807-34744 | 2000 | female | Landmark on the Lake | Wisconsin |
| 1807-77645 | 2000 | female | Colonnade | Minnesota |
| 1807-77646 | 2000 | female | Colonnade | Minnesota |
| 1807-77647 | 2000 | male | Colonnade | Minnesota |
| 1807-77672 | 2000 | female | River | Illinois |
| 1807-77673 | 2000 | female | Wacker | Illinois |
| 1807-77674 | 2000 | female | Wacker | Illinois |
| 1807-77675 | 2000 | female | Hyde Park | Illinois |
| 1807-91943* | 2000 | female | Woodmen Tower | Nebraska |
| 2206-28983 | 2000 | male | River | Illinois |
| 2206-28984 | 2000 | male | River | Illinois |
| 2206-28985 | 2000 | male | UIC | Illinois |
| 2206-28986 | 2000 | male | UIC | Illinois |
| 2206-28987 | 2000 | male | Wacker | Illinois |
| 2206-28988 | 2000 | male | Wacker | Illinois |
| 2206-28989 | 2000 | male | Hyde Park | Illinois |
| 2206-28990 | 2000 | male | Hyde Park | Illinois |
| 2206-28991 | 2000 | male | Hyde Park | Illinois |
| 2206-62723 | 2000 | male | North Central Life | Minnesota |
| 1807-34717 | 2001 | female | Terminal Tower | Ohio |
| 1807-34718 | 2001 | female | Terminal Tower | Ohio |
| 1807-69740 | 2001 | female | Uptown | Illinois |
| 1807-69757 | 2001 | female | Wacker | Illinois |
| 1807-77651 | 2001 | female | Colonnade | Minnesota |
| 1807-77652 | 2001 | female | Colonnade | Minnesota |
| 1807-77653 | 2001 | male | Colonnade | Minnesota |
| 1807-77677 | 2001 | female | UIC | Illinois |
| 1807-77678 | 2001 | female | UIC | Illinois |
| 1807-77683 | 2001 | female | UIC | Illinois |
| 2206-28933 | 2001 | male | Colonnade | Minnesota |
| 2206-35789 | 2001 | male | NIPSCO Plant | Indiana |
| 2206-41028 | 2001 | male | Wacker | Illinois |
| 2206-41030 | 2001 | male | Wacker | Illinois |
| 2206-41031 | 2001 | male | Wacker | Illinois |
| 2206-41059 | 2001 | male | Wacker | Illinois |
| 2206-62735 | 2001 | male | Terminal Tower | Ohio |
| 2206-62736 | 2001 | male | Terminal Tower | Ohio |
| 2206-62822 | 2001 | male | UIC | Illinois |
| 2206-62828 | 2001 | male | Uptown | Illinois |
| 2206-62829 | 2001 | male | Uptown | Illinois |
| 2206-62831 | 2001 | male | Prison | Illinois |
| 1807-34720* | 2002 | male | One Summit Square | Indiana |
| 1807-35921* | 2002 | female | Cleveland Clinic | Ohio |
| 1807-35941* | 2002 | female | Hilliard Road Bridge | Ohio |
| 1807-35942* | 2002 | female | I-90 Bridge | Ohio |
| 1807-35943 | 2002 | female | Colonnade | Minnesota |
| 1807-35945* | 2002 | female | Palisade Head | Minnesota |
| 1807-35950* | 2002 | female | Ford Parkway | Minnesota |
| 1807-49799 | 2002 | female | Landmark on the Lake | Wisconsin |
| 1807-62101* | 2002 | female | Castle Cliff | Minnesota |
| 1807-69727* | 2002 | female | WPS Pulliam Power Plant | Wisconsin |
| 1807-69735 | 2002 | female | UIC | Illinois |
| 1807-69739 | 2002 | female | UIC | Illinois |
| 1807-77679 | 2002 | female | River | Illinois |
| 1807-77680 | 2002 | female | Pilsen | Illinois |
| 1807-77681 | 2002 | female | Uptown | Illinois |
| 1807-77682 | 2002 | male | Uptown | Illinois |
| 1807-77789 | 2002 | female | Terminal Tower | Ohio |
| 1807-77796 | 2002 | female | Hyde Park | Illinois |
| 1807-77797 | 2002 | female | Waukegan | Illinois |
| 1807-91912* | 2002 | female | NIPSCO Bailly Plant | Indiana |
| 1807-91915* | 2002 | female | U.S. Steel | Indiana |
| 1807-91916* | 2002 | female | Kokomo Gas and Power | Indiana |
| 1807-91965* | 2002 | female | MidAmerican Energy | Iowa |
| 2206-47669 | 2002 | male | Wacker | Illinois |
| 2206-47670 | 2002 | male | Wacker | Illinois |
| 2206-47671* | 2002 | male | First Bank | Iowa |
| 2206-47672* | 2002 | male | MEC Louisa | Iowa |
| 2206-62715* | 2002 | male | Market Tower | Indiana |
| 2206-62749* | 2002 | male | Queens Bluff | Minnesota |
| 2206-62793* | 2002 | male | Corundum Point | Minnesota |
| 2206-62797* | 2002 | male | Kewaunee Nuclear Power Plant | Wisconsin |
| 2206-62820* | 2002 | male | Daniel Boone National Forest | Kentucky |
| 2206-62823 | 2002 | male | UIC | Illinois |
| 2206-62824 | 2002 | male | River | Illinois |
| 2206-62825 | 2002 | male | Waukegan | Illinois |
| 2206-62826 | 2002 | male | Waukegan | Illinois |
| 2206-62827 | 2002 | male | Waukegan | Illinois |
| 2206-62847* | 2002 | male | Miami Fort Station, Cleves | Ohio |
| 2206-62851 | 2002 | male | Terminal Tower | Ohio |
| 2206-62852 | 2002 | male | Terminal Tower | Ohio |
| 2206-62853 | 2002 | male | Terminal Tower | Ohio |
| 2206-62854* | 2002 | male | Ironton/Russell Bridge | Ohio |
| 2206-62860* | 2002 | male | First Merit Bank | Ohio |
| 2206-62861 | 2002 | male | Chemed Center | Ohio |
| 2206-62882 | 2002 | male | Colonnade | Minnesota |
| 2206-62885* | 2002 | male | Faith Bluff | Minnesota |
| 2206-69819* | 2002 | male | Riverside Plaza/I-94 Mississippi River | Minnesota |
| FMNH-508008^1^ | 2002 | male | Etobicoke | Ontario |
| 1687-01961 | 2003 | female | Waukegan | Illinois |
| 1807-62131 | 2003 | female | IPL Stout Plant | Indiana |
| 1807-62177 | 2003 | female | Colonnade | Minnesota |
| 2206-49404 | 2003 | male | Colonnade | Minnesota |
| 2206-49420 | 2003 | male | WEPCO Power Plant | Wisconsin |
| 2206-49427 | 2003 | male | MB(WI) | Wisconsin |
| 2206-49431* | 2003 | male | Froedtert Malt | Wisconsin |
| 987-40120 | 2003 | female | Pilsen | Illinois |
| 987-40220 | 2003 | female | WPL Edgewater | Wisconsin |
| 1807-62141 | 2004 | female | First Bank | Iowa |
| 1807-91957 | 2004 | female | Waukegan | Illinois |
| 2206-49446 | 2004 | male | Waukegan | Illinois |
| 2206-49447 | 2004 | male | Waukegan | Illinois |
| 2206-49448 | 2004 | male | Waukegan | Illinois |
| 2206-49449 | 2004 | male | Pilsen | Illinois |
| 2206-49450 | 2004 | male | Wacker | Illinois |
| 2206-49451 | 2004 | male | Broadway | Illinois |
| 2206-49453 | 2004 | male | Wacker | Illinois |
| 2206-62892 | 2004 | male | Uptown | Illinois |
| 987-40121 | 2004 | female | Uptown | Illinois |
| 987-40122 | 2004 | female | Uptown | Illinois |
| 987-40123 | 2004 | female | Uptown | Illinois |
| 987-40124 | 2004 | female | Pilsen | Illinois |
| 987-40125 | 2004 | female | Wacker | Illinois |
| 987-40126 | 2004 | female | Wacker | Illinois |
| 987-40127 | 2004 | female | Prison | Illinois |
| 987-40272 | 2004 | female | Pilsen | Illinois |
| 987-40273 | 2004 | female | Broadway | Illinois |
| 987-40274 | 2004 | female | Evanston | Illinois |
| 987-40275 | 2004 | female | Evanston | Illinois |
| 987-40299 | 2004 | female | J.M. Stuart Power Plant | Ohio |
| 1687-01957 | 2005 | female | Evanston | Illinois |
| 1687-01958 | 2005 | female | Evanston | Illinois |
| 1687-01959 | 2005 | female | Evanston | Illinois |
| 1687-01960 | 2005 | female | River | Illinois |
| 2206-47674 | 2005 | male | Uptown | Illinois |
| 2206-47680 | 2005 | male | Prison | Illinois |
| 2206-49452 | 2005 | male | Pilsen | Illinois |
| 2206-62794 | 2005 | male | Waukegan | Illinois |
| 2206-62895 | 2005 | male | Broadway | Illinois |
| 2206-62896 | 2005 | male | Broadway | Illinois |
| 2206-62897 | 2005 | male | Pilsen | Illinois |
| 2206-62899 | 2005 | male | Uptown | Illinois |
| 2206-62900 | 2005 | male | Uptown | Illinois |
| 987-40278 | 2005 | female | Waukegan | Illinois |
| 987-40279 | 2005 | female | Waukegan | Illinois |
| 987-40280 | 2005 | female | Waukegan | Illinois |
| 987-40281 | 2005 | female | Broadway | Illinois |
| 987-40282 | 2005 | female | Broadway | Illinois |
| 987-40283 | 2005 | female | Pilsen | Illinois |
| 987-40284 | 2005 | female | Broadway | Illinois |
| 987-40285 | 2005 | female | Pilsen | Illinois |
| 987-40286 | 2005 | female | Prison | Illinois |
| 987-40287 | 2005 | female | Waukegan | Illinois |
| 1687-01710 | 2006 | female | WPL Edgewater | Wisconsin |
| 1687-01711 | 2006 | female | WPL Edgewater | Wisconsin |
| 1687-01825 | 2006 | female | Cargill Malt Complex | Wisconsin |
| 1687-01829* | 2006 | female | State Capitol | Nebraska |
| 1687-01835* | 2006 | female | Woodmen Tower | Nebraska |
| 1687-02008* | 2006 | female | AT&T Building | Ohio |
| 1687-02018 | 2006 | female | Pilsen | Illinois |
| 1687-02021 | 2006 | female | Waukegan | Illinois |
| 1687-02022 | 2006 | female | Waukegan | Illinois |
| 1687-02023 | 2006 | female | Saint Michael's | Illinois |
| 1687-02024 | 2006 | female | Saint Michael's | Illinois |
| 1687-02025 | 2006 | female | Wacker | Illinois |
| 1687-02026 | 2006 | female | Broadway | Illinois |
| 1687-02027 | 2006 | female | Broadway | Illinois |
| 1687-02028 | 2006 | female | Evanston | Illinois |
| 1687-02031 | 2006 | female | Prison | Illinois |
| 1687-02081* | 2006 | female | First National Bank | Ohio |
| 1687-02091* | 2006 | female | Bank One | Ohio |
| 1687-02102 | 2006 | female | Cargill Malt Complex | Wisconsin |
| 2206-49470 | 2006 | male | Pilsen | Illinois |
| 2206-49471 | 2006 | male | Waukegan | Illinois |
| 2206-49472 | 2006 | male | Waukegan | Illinois |
| 2206-49473 | 2006 | male | Saint Michael's | Illinois |
| 2206-49474 | 2006 | male | Wacker | Illinois |
| 2206-72234* | 2006 | male | Commodore Perry Motor Inn | Ohio |
| 2206-72281* | 2006 | male | Bohn Building | Ohio |
| 2206-72293 | 2006 | male | Wacker | Illinois |
| 2206-72297 | 2006 | male | Wacker | Illinois |
| 2206-72298 | 2006 | male | Broadway | Illinois |
| 2206-72300 | 2006 | male | Evanston | Illinois |
| 2206-72361 | 2006 | male | Broadway | Illinois |
| 2206-72362 | 2006 | male | Uptown | Illinois |
| 2206-72363 | 2006 | male | Uptown | Illinois |
| 2206-72365 | 2006 | male | Pilsen | Illinois |
| 2206-72400 | 2006 | male | Evanston | Illinois |
| 2206-84521 | 2006 | male | WPL Edgewater | Wisconsin |
| 2206-84538 | 2006 | male | WPL Edgewater | Wisconsin |
| 1687-01775 | 2007 | female | Prison | Illinois |
| 1687-01776 | 2007 | female | Evanston | Illinois |
| 1687-01834 | 2007 | female | Wacker | Illinois |
| 1687-01839 | 2007 | female | Wacker | Illinois |
| 1687-01840 | 2007 | female | Broadway | Illinois |
| 1687-01896 | 2007 | female | UIC | Illinois |
| 1687-01900 | 2007 | female | Pilsen | Illinois |
| 1687-02068 | 2007 | female | Waukegan | Illinois |
| 1687-02101 | 2007 | female | Cargill Malt Complex | Wisconsin |
| 1687-02103 | 2007 | female | Cargill Malt Complex | Wisconsin |
| 2206-84626 | 2007 | male | Broadway | Illinois |
| 2206-84627 | 2007 | male | Broadway | Illinois |
| 2206-84628 | 2007 | male | Waukegan | Illinois |
| 2206-84629 | 2007 | male | Waukegan | Illinois |
| 2206-84630 | 2007 | male | Uptown | Illinois |
| 2206-84631 | 2007 | male | Uptown | Illinois |
| 2206-84632 | 2007 | male | Uptown | Illinois |
| 2206-84633 | 2007 | male | Pilsen | Illinois |
| 2206-84634 | 2007 | male | UIC | Illinois |
| 2206-84635 | 2007 | male | UIC | Illinois |
| 2206-84636 | 2007 | male | Evanston | Illinois |
| 2206-84637 | 2007 | male | Evanston | Illinois |
| 2206-84638 | 2007 | male | Broadway | Illinois |
| 2206-84639 | 2007 | male | Evanston | Illinois |
| 2206-84678 | 2007 | male | Cargill Malt Complex | Wisconsin |
| 1126-06404 | 2008 | male | Waukegan | Illinois |
| 1126-06406 | 2008 | male | Waukegan | Illinois |
| 1687-21400 | 2008 | female | Broadway | Illinois |
| 1687-21401 | 2008 | female | Broadway | Illinois |
| 1687-21402 | 2008 | female | Waukegan | Illinois |
| 1687-21403 | 2008 | female | Waukegan | Illinois |
| 1687-21404 | 2008 | female | Waukegan | Illinois |
| 1687-21405 | 2008 | female | Wacker | Illinois |
| 1687-21406 | 2008 | female | UIC | Illinois |
| 1687-21407 | 2008 | female | UIC | Illinois |
| 1687-21408 | 2008 | female | UIC | Illinois |
| 1687-21409 | 2008 | female | Prison | Illinois |
| 1687-21422 | 2008 | female | Evanston | Illinois |
| 1687-21423 | 2008 | female | Evanston | Illinois |
| 1687-21424 | 2008 | female | Evanston | Illinois |
| 816-38648 | 2008 | male | Broadway | Illinois |
| 816-38649 | 2008 | male | Evanston | Illinois |
| 816-38650 | 2008 | male | Wacker | Illinois |
| 1126-14001 | 2009 | male | Waukegan | Illinois |
| 1126-14002 | 2009 | male | Evanston | Illinois |
| 1126-14003 | 2009 | male | Broadway | Illinois |
| 1126-14004 | 2009 | male | Broadway | Illinois |
| 1126-14005 | 2009 | male | Uptown | Illinois |
| 1126-14006 | 2009 | male | Uptown | Illinois |
| 1126-14007 | 2009 | male | Uptown | Illinois |
| 1126-14008 | 2009 | male | Wacker | Illinois |
| 1126-14009 | 2009 | male | Wacker | Illinois |
| 1126-14010 | 2009 | male | UIC | Illinois |
| 1126-14011 | 2009 | male | UIC | Illinois |
| 1126-14012 | 2009 | male | South Loop | Illinois |
| 1687-30321 | 2009 | female | Waukegan | Illinois |
| 1687-30322 | 2009 | female | Waukegan | Illinois |
| 1687-30323 | 2009 | female | Waukegan | Illinois |
| 1687-30324 | 2009 | female | Evanston | Illinois |
| 1687-30325 | 2009 | female | Evanston | Illinois |
| 1687-30326 | 2009 | female | Evanston | Illinois |
| 1687-30327 | 2009 | female | Broadway | Illinois |
| 1687-30328 | 2009 | female | Broadway | Illinois |
| 1687-30329 | 2009 | female | Broadway | Illinois |
| 1687-30330 | 2009 | female | Uptown | Illinois |
| 1687-30331 | 2009 | female | Wacker | Illinois |
| 1687-30332 | 2009 | female | Wacker | Illinois |
| 1687-30333 | 2009 | female | South Loop | Illinois |
| 1687-30334 | 2009 | female | South Loop | Illinois |

**Table B in S1 File.** **Detailed contribution from field data and genetic data on the kinship relationships for all Midwestern surveyed nests.**

| Nest | Evidence from Field Data | | Evidence from Genetic Data |
| --- | --- | --- | --- |
| Chicago area (IL) | | | |
| Broadway | 1994-2003: 95T (male), 2/8 (female). 2004-2009: *P/M (male), 5/*P (female) but male for 2009 was not identified | | Confirmed field observations for 1994-98 and 2004-09. No samples for the period 1999-2003. New male confirmed in 2009. |
| Evanston | 1999-2002: a pair of birds seen intermittently. No nesting in 2003. 2004-2009: One male (P/N) identified on 2004-05, no ID for male on 2006, 48/M during 2007-09. Female 1/*D seen only in 2004, new female (64/D) in 2005-09 | | Confirmed field observations. Evidence that new male on 2006 was neither the predecesor nor the successor. |
| Hyde Park | 1994-2002: Presumably same male (77T) and female (22R) nesting all these years. IDs not always available as birds changed location of nest several times. 2003-09: Same female (22R) observed pairing with son (S/T) | | Sibship analyses confirmed same breeding pair for the period 1994-2002. Because offspring not produced since 2003 we could not confirm mother (22R)-offspring (S/T) pairing. |
| Pilsen | 2002-07: Male (L/N) presumably nesting with female (9/*Y) but they were not positively ID each year. No offspring in 2008. New female (R/38) in 2009. | | Sibship analyses confirmed same breeding pair for the period 2002-07. Because offspring not produced in 2009 we could not confirm new female (R/38). |
| Prison | No parent sampled. Sampling from chicks is done when chicks get grounded in first flight. 1998-2003: Parents banded, and no chicks sampled in 2000, 2002-03. 2004-2005: Presumably same male (5/*E) and female (*D/W), no chicks sampled on 2003 and 2009. Only one or two chicks sampled in remaining years. | | Sibship analysis show full sibs in any given year. Half-sibs between 2005-06 indicating replacement of one of the parents. |
| Saint Michael | 2005: Male (58/M), female (75/E), produced 3 unsampled chicks. 2006: same male, female (60/D), produced 4 chicks, 3 sampled. 2007-08: same male, female (69/C), no chicks. 2009: unbanded male, same female, no chicks. | | Male (58/M), females (75/E & 60/D) were genotyped and parentage confirmed for 2006. |
| River | Eight different locations reported for this nest, making it difficult for sampling. 1998-2002: Presumably same male (88Y) and same female (62V). No chicks on 2003, one chick unbanded in 2004. 2005-07: Male unknown, female (01/A) produced only one chick. 2008-09: Male unknown, female (V/S), they had no chicks. | Sibship analysis show full sibs for 1998-2002. Parent's genotypes reconstructed. Mother in 2005 (01/A) was genotyped and only chick confirmed to be hers. | |
| UIC | 1999-2004: Presumable male (0/*A), female (*6/D), 18 chicks produced, 12 sampled. 2005-09: different unbanded male, same female. No chicks sampled in 2005-06. Eight chicks sampled during 2007-09. | Parentage confirmed for 1999-2004. No sampling for 2005-06. Sibships analysis show for period 2007-09 that two different unbanded males sired the chicks. One male in 2007 and a different male in 2008-09. | |
| Uptown | 2001: Unbanded male and dubious female (*H/4 or *4/H?). 2002: unbanded male, female (*4/H). 2003-09: male (G/G), same female. They produced | Parentage analysis confirms that female *4/H (not *H/4) was the mother in 2001 and it has been breeding there until 2009. Two males have breed on the same nest: one unbanded (2001-02) and G/G (2003-09). | |
| Wacker | 1987-1991: Male (Jingles) paired with female (*P/5) were observed intermittently during those years. During that period, three chicks were not sampled. 1992-2004: Presumably male (Hubert) and one female (*P/*5, until 1997), another female (E/E, 1998-99), and last female (*C/4, 2000-04). Thirty-seven chicks produced, 28 sampled. No nesting in 2005. 2006-07: Male (7/6) with female (01/A) produced six chicks. 2008-09: Male (59/H) with same female also produced six chicks. | Not genetic data for 1987-1991. Parental reconstruction sibship analysis allowed the inference of the genotype of male (Hubert) and confirmed nesting for the period 1993-2004. Evidence of EPF in 1998 and presence of new female (E/E). The two other females (*P/*5 & *C/4) were confirmed as breeders for the same period. For period 2006-09, breeders were also confirmed through parentage analysis. | |
| Waukegan | 2001-2009: Male is banded but no further info available paired with female (5/*X) produced 37 chicks from which 30 were sampled. | Parental reconstruction and sibship analyses showed two males were nesting at this site. One male during 2002-06 and a second male during 2007-09. Enough offspring allowed for the genotypic reconstruction of both males. | |
| South Loop | 2009: Male unbanded with female (79/D) produced three chicks | Sibship analysis showed that this is a full-sib group. | |
|  | | | |
| Detroit (MI) | | | |
| Whittier | 1997: Male unbanded with female (55R) produced one chick (*5/C). In 1999 this chick and same female (his mother) produced 2 chicks but samples were unavailable. | Mother-offspring relationship confirmed. Inbreeding could not be tested due to lack of offspring samples. | |
| Minneapolis, St. Paul, Monticello (MN) | | | |
| Colonnade | 1993-2003: Male (31T) paired with different females throughout this period. Female (81V, 1992-94), unbanded female (1995), female (Elma, 1996-98), female (*4/C, 1999-2003). Thirty-three offspring were produced from which 31 were sampled. | Male (31T) was confirmed and his genotype reconstructed by parentage and sibship analysis. Genotypes from females (81V & Elma) were also reconstructed. Last female (*4/C) was confirmed as breeding during 1999-03. | |
| Riverside | 1998: Unknown male was paired with female (K/*V) and produced two chicks | Mother-offspring relationship confirmed. Cannot reconstruct the father's genotype due to lack of sampling | |
| NSP High Bridge | 1999: Male (04T) paired with female (12R) and produced four chicks. This male was previously a nester in North Central Life, MN (1996-97) | Mother-offspring relationship confirmed. Father's genotype reconstructed. | |
| NSP Monticello | 1997: Unknown male with female (K/*V) produced four chicks and three were sampled. | Mother-offspring relationship confirmed. Cannot reconstruct the father's genotype due to lack of sampling. | |
| Cleveland (OH) | | | |
| Terminal Tower | 2001-02: Male (*R/K) paired with female (23W) in 2001 and female (S/*W) in 2002. They produced eight chicks. | Parentage confirmed for 2001. Parentage and sibship analysis allowed for mother's genotype reconstruction in 2002. | |
| Jefferson-Milwaukee-Sheboygan (WI) | | | |
| Cargill Malt | 2006-07: Unknown male paired with female (82/A) produced 5 chicks. | Mother-offspring relationship confirmed. Father's genotype reconstructed. | |
| Froedtert Malt | 2003: Male (C/D) paired with female (A/*H) produced one chick. | Mother-offspring relationship confirmed. Cannot reconstruct the father's genotype due to lack of sampling. | |
| Landmark Lake | 1999-2002: Male (76T) paired with female (U/K) produced three chicks | Sibship analysis shows one full-sib group. Parent's genotypes cannot be reconstructed due to lack of sampling | |
| WPL Edgewater | 1999-2003: Banded male paired with female (E/*D) producing two chicks. No sampled chicks during 2004-05. 2006: Banded male paired with same female produced 4 chicks. | Sibship analysis shows two full-sib groups. Parent's genotypes cannot be reconstructed due to lack of sampling | |
